# Development of a Methodology for Low-Cost 3D Underwater Motion Capture: Application to the Biomechanics of Horse Swimming

**DOI:** 10.3390/s23218832

**Published:** 2023-10-30

**Authors:** Chloé Giraudet, Claire Moiroud, Audrey Beaumont, Pauline Gaulmin, Chloé Hatrisse, Emeline Azevedo, Jean-Marie Denoix, Khalil Ben Mansour, Pauline Martin, Fabrice Audigié, Henry Chateau, Frédéric Marin

**Affiliations:** 1 Laboratoire de BioMécanique et BioIngénierie (UMR CNRS 7338), Centre of Excellence for Human and Animal Movement Biomechanics (CoEMoB), Université de Technologie de Compiègne (UTC), Alliance Sorbonne Université, 60200 Compiègne, France; chloe.giraudet@utc.fr (C.G.); khalil.ben-mansour@utc.fr (K.B.M.); 2 CIRALE, USC 957 BPLC, Ecole Nationale Vétérinaire d’Alfort, 94700 Maisons-Alfort, France; claire.moiroud@vet-alfort.fr (C.M.); audrey.beaumont@vet-alfort.fr (A.B.); pauline.gaulmin@vet-alfort.fr (P.G.); chloe.hatrisse@vet-alfort.fr (C.H.); jean-marie.denoix@vet-alfort.fr (J.-M.D.); henry.chateau@vet-alfort.fr (H.C.); 3 Univ Lyon, Univ Gustave Eiffel, Univ Claude Bernard Lyon 1, LBMC UMR_T 9406, 69622 Lyon, France; 4 LIM France, Chemin Fontaine de Fanny, 24300 Nontron, France

**Keywords:** underwater motion capture, 3D reconstruction, horse swimming, joint angles

## Abstract

Hydrotherapy has been utilized in horse rehabilitation programs for over four decades. However, a comprehensive description of the swimming cycle of horses is still lacking. One of the challenges in studying this motion is 3D underwater motion capture, which holds potential not only for understanding equine locomotion but also for enhancing human swimming performance. In this study, a marker-based system that combines underwater cameras and markers drawn on horses is developed. This system enables the reconstruction of the 3D motion of the front and hind limbs of six horses throughout an entire swimming cycle, with a total of twelve recordings. The procedures for pre- and post-processing the videos are described in detail, along with an assessment of the estimated error. This study estimates the reconstruction error on a checkerboard and computes an estimated error of less than 10 mm for segments of tens of centimeters and less than 1 degree for angles of tens of degrees. This study computes the 3D joint angles of the front limbs (shoulder, elbow, carpus, and front fetlock) and hind limbs (hip, stifle, tarsus, and hind fetlock) during a complete swimming cycle for the six horses. The ranges of motion observed are as follows: shoulder: 17 ± 3°; elbow: 76 ± 11°; carpus: 99 ± 10°; front fetlock: 68 ± 12°; hip: 39 ± 3°; stifle: 68 ± 7°; tarsus: 99 ± 6°; hind fetlock: 94 ± 8°. By comparing the joint angles during a swimming cycle to those observed during classical gaits, this study reveals a greater range of motion (ROM) for most joints during swimming, except for the front and hind fetlocks. This larger ROM is usually achieved through a larger maximal flexion angle (smaller minimal angle of the joints). Finally, the versatility of the system allows us to imagine applications outside the scope of horses, including other large animals and even humans.

## 1. Introduction

Being able to accurately perform three-dimensional motion capture (3D MoCap) of a body underwater holds significance in understanding swimming biomechanics. In the context of human swimming performance, the ability to reconstruct 3D motion would greatly contribute to the understanding of the subtle variations among swimmers that can ultimately lead to improved speed and efficiency [1]. In the case of equine science, the swimming performance of the horse may not be the primary concern. Hydrotherapy has been employed for over four decades, and its benefits have been proven for rehabilitation [2]. However, in the context of equine hydrotherapy, understanding and monitoring the movements involved in a swimming cycle are crucial for effective rehabilitation after an injury, particularly the control of the joint range of motion (ROM) [3].

For humans and animals, 3D MoCap is a standard research method in biomechanics [4]. Even though the techniques of 3D MoCap for terrestrial locomotion are well established, applying 3D MoCap in the aquatic environment still poses a challenge. Traditional methods for motion capture systems can be categorized into three primary types: optical marker-based systems, sensor-based systems, and optical marker-less systems [5,6,7]. Optical marker-based systems, often considered the gold standard, involve the use of spherical external markers glued to anatomical landmarks and cameras. Such systems tend to be expensive due to the number of cameras required and are time-consuming to install if not inside a fixed laboratory setup [8,9]. In the aquatic environment, additional challenges arise, such as waterproofing the cameras and dealing with the drag induced by external spherical markers [10,11]. Sensor-based systems, which rely on Inertial Measurement Units (IMUs), have gained popularity due to advancements in technology [12]. However, in the aquatic environment, additional drag is expected due to the size of the sensors, and specific post-processing methods and analysis techniques are required to accurately quantify the motion of horses [13]. It is important to note that these methods have not yet been validated specifically for the aquatic environment. Marker-less systems can take various forms, including manual digitization (using software such as Kinovea (https://www.kinovea.org/, accessed on 7 November 2022), depth cameras [14,15], or deep neural networks [16]. However, these approaches also have their own limitations. Manual digitization and depth cameras can be time-consuming in terms of post-processing or setup requirements, respectively. Additionally, while deep neural networks are being developed for human motion capture [17,18] and terrestrial horse motion capture [19,20], their application to underwater horse swimming remains unavailable and presents a significant challenge, primarily because there are no training data available.

To develop a system capable of 3D tracking anatomical segments under aquatic conditions, two criteria must be met. Firstly, non-invasiveness is essential to faithfully reproduce the natural movement, ensuring that the acquisition system does not introduce any additional drag or resistance [21]. Secondly, accuracy is contingent upon the measurement range of motion and can only be evaluated by examining specific joints [22]. Non-invasiveness poses significant challenges in equine science, primarily due to two reasons. Firstly, horses are sensitive animals that are averse to changes in their environment [23]. As a result, the system must be discreet and minimize disruptions to ensure the horse’s comfort. Secondly, a swimming pool is typically used for the conventional training and rehabilitation of clients’ horses, requiring the motion capture system to operate without interfering with the pool’s regular usage. Regarding accuracy, it should be determined by the range of motion exhibited by the horse’s joints when swimming in the pool. The precise measurement and analysis of joint motion within this aquatic context are crucial for gaining insights into the rehabilitative process. Finally, the available space in a horse swimming pool is limited, typically with a maximum width of only three meters. Considering that horses are relatively large animals, capturing the entire swimming cycle requires a system capable of acquiring a substantial volume (measured in cubic meters) while remaining compact.

To the best of our knowledge, only one study has been conducted on the underwater 2D movement of horses [24,25]. However, the research did not comprehensively describe the swimming cycle. Specifically, the authors examined the movements of the front and hind limbs independently, which hindered the ability to determine the necessary anterior–posterior and lateral–lateral coordination for defining the spatial-temporal parameters. Additionally, they did not evaluate the precision of the measurements. Nonetheless, their work provided initial insights into the expected range of motion for different joints in three dimensions and underscored the required accuracy for each joint.

In this study, our aim was to develop an affordable, non-invasive, and user-friendly marker-based capture system for analyzing the 3D movement of horses throughout an entire swimming cycle. The system we developed involves the utilization of waterproof cameras and surface markers drawn on the horse, along with the necessary pre- and post-processing techniques to reconstruct and analyze the captured data. We expect to determine joint angles with a precision of 1° and also confirm a range of motion larger than that on land for all joints, except for the fetlocks, similar to what was found in [24,25].

## 2. Materials and Methods

In this study, the computation of the horse’s 3D reconstruction during a swimming cycle was achieved through a process divided into three main steps:

1. Video Acquisition: The first step involved capturing the necessary video footage of the horse during its swimming cycle.
2. Video Pre-Processing: After video acquisition, the obtained footage underwent pre-processing to enhance the quality and its suitability for subsequent analysis. This pre-processing step involved tasks such as calibration, synchronization, marker tracking, and any necessary adjustments to ensure optimal data accuracy.
3. Video Post-Processing: The final step encompassed the post-processing of the pre-processed video data. This involved employing specialized algorithms to accurately reconstruct the horse’s 3D movement.

### 2.1. Video Acquisition

#### 2.1.1. Acquisition System

**Figure 1:**
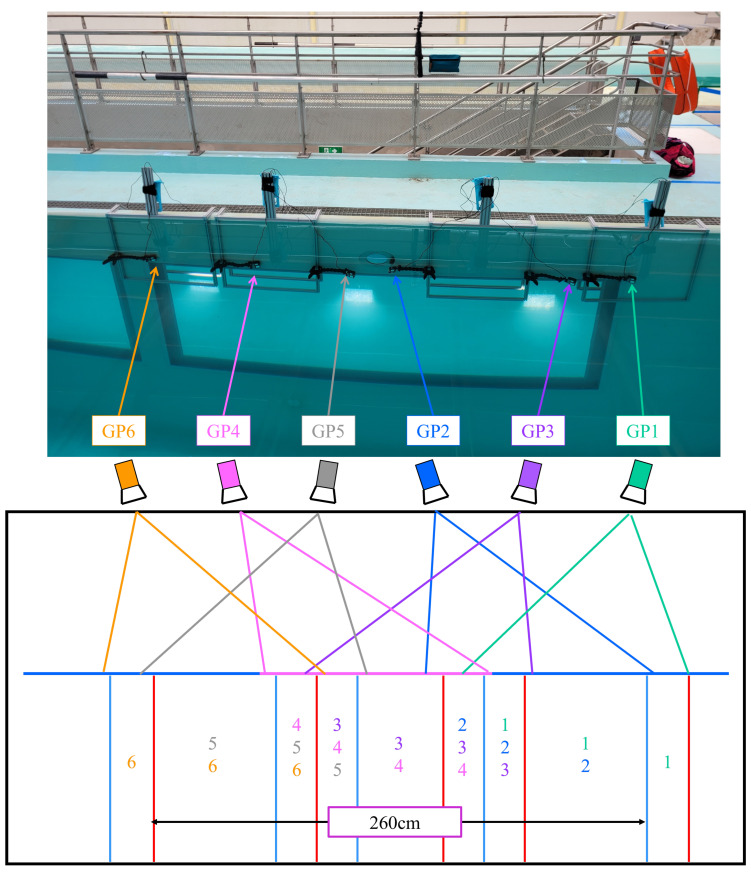
Acquisition system and field of view. **Top**: The six cameras (GP = GoPro) are placed on homemade supports in front of the window (in the swimming pool) and illuminated by spotlights positioned behind the window. **Bottom**: Schematized top view of the placement and field of view for each camera. A 2.6-m field of view is achieved with overlapping fields of view for each pair of cameras.

The acquisition system should meet the following requirements: (1) be waterproof to eliminate the issue of a glass window, (2) have a large field of view to enable filming the entire body of the horse with minimal distance between the camera and the horse, (3) be capable of filming at a high-speed rate to capture the rapid movement of the horse’s limbs, and (4) have a sufficient battery capacity to film an entire swimming session. Our choice was to use six waterproof cameras (GoPro Hero 8 Black, GoPro, San Mateo, USA) [9], which were controlled using smartphones. These cameras were positioned to capture six different views of the horse underwater. The cameras operated at a high frame rate of 120 frames per second and a resolution of 2.7 K. The cameras were fixed on a modular aluminum structure (V-SLOT 20 × 20 slot 6 mm https://www.systeal.com/fr/profiles-aluminium-6-mm/1221-profile-aluminium-v-slot-20x20-fente-6-mm.html (accessed on 24 January 2023), SystéAl, Lognes, France) on one side of the swimming pool (see Figure 1—top). The localization of these cameras allowed for a field-of-view measurement of approximately 2.6 m in length and 3.0 m in height (see Figure 1—bottom). Additional lighting was provided by three spotlights positioned behind the underwater window of the swimming pool (see Figure 1—top). It should be noted that while the horses were capable of swimming in both directions (right or left side), only the side of the horse facing the camera (the side in front of the camera) was recorded.

**Figure 2:**
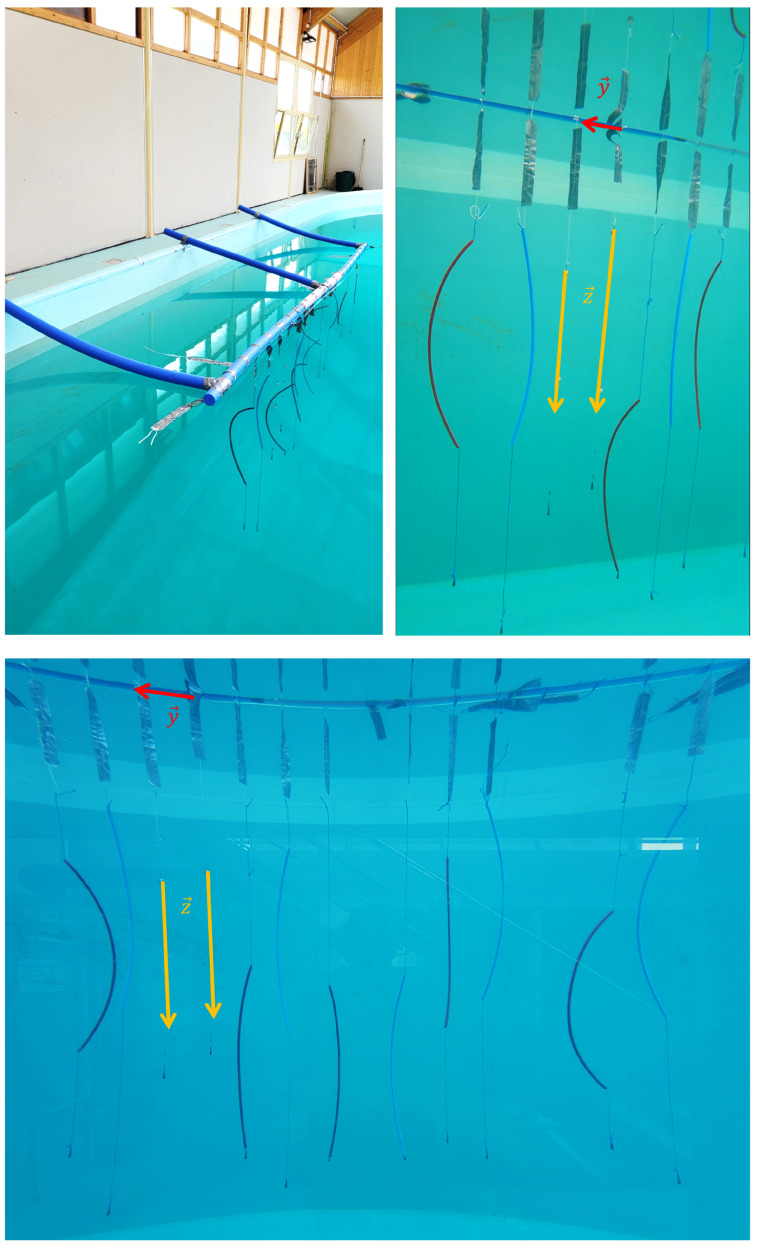
Phantom made of foam (swimming foam fries) with plumber pipes attached to target specific fields of view for each camera. **Top left**: View from the top. **Top right**: View from GP1, the first GoPro camera on the left. **Bottom**: View from the front through the window of the swimming pool. The distortion induced by the glass can be observed in the bottom border of the swimming pool, which should appear straight.

The cameras’ localizations and orientations were tuned using a foam phantom (Figure 2). The foam phantom served as a reference for determining the camera directions and helped establish the coordinate system for the swimming pool. To establish the coordinate system, fluorescent balls were attached to fishing lines weighted with fishing weights. These balls served as markers to define the vertical direction (z-axis, depicted in yellow in the image on the right in Figure 2). Additionally, foam fries were utilized to delineate the horizontal plane of the swimming pool (y-axis, depicted in red in the image on the right in Figure 2), aligning with the waterline.

#### 2.1.2. Horses

**Figure 3:**
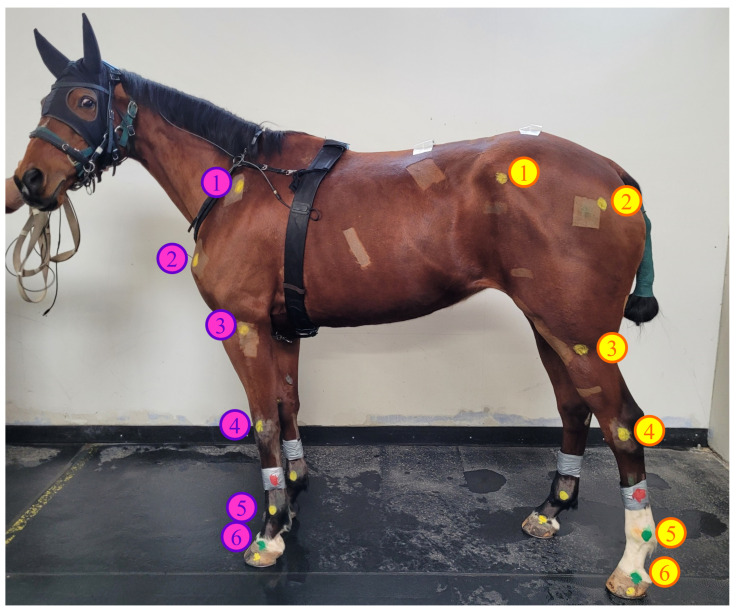
Anatomical points painted on the horse. Six points are marked on each front and hind limb of the horse, as shown in Table 1. Pink points represent the forelimb, while yellow points represent the hind limb.

For this study, six jumping horses were included (see Table A1). The rationale for the size sample was that the number of six individuals was expected to provide sufficient statistical power for biomechanical joint angle analysis [26]. The horses underwent a three-month training protocol designed by the veterinary team. During the first month, the horses followed a conventional training regimen, whereas in the last two months, three swimming sessions were included as part of their training routine. Throughout these final two months, the swimming sessions were recorded using the previously described acquisition system (refer to Section 2.1.1 for details). On each horse, 24 anatomical markers were applied by the veterinary team [27] using cattle markers (animal marking sticks https://www.raidex.de/en/products/animal-marking-sticks (accessed on 2 December 2022), Raidex, Dettingen, Germany). The markers used in this study were disc-shaped with a radius of 25 mm. These markers were chosen to have contrasting colors compared to the horse’s coat color (in this case, yellow markers were used on a bay horse, as shown in Figure 3). The markers were placed at six specific anatomical locations on each limb, as outlined in Table 1. For the front limb, the marker locations included the scapula, shoulder, elbow, carpus, fetlock, and hoof (Figure 3). For the hind limb, the marker locations included the tuber coxae, hip, stifle, tarsus, fetlock, and hoof (Figure 3).

This study was approved by the clinical research and ethics committee of the Alfort Veterinary School (protocol code: 2022-09-19; date of approval: 14 November 2022).

### 2.2. Video Pre-Processing

**Figure 4:**
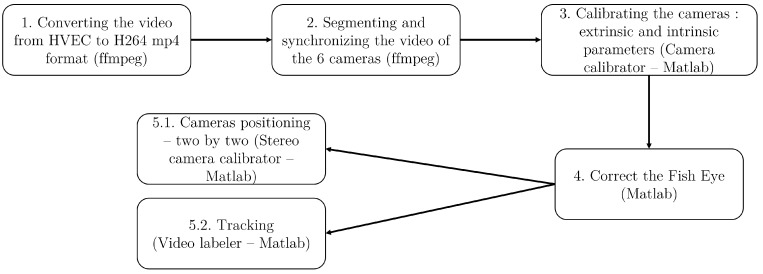
The five steps involved in pre-processing the videos from the 6 GoPro cameras.

The pre-processing steps required to reconstruct the 3D coordinates of the anatomical points from the videos are outlined in Figure 4. These steps can be summarized as follows:

1. Conversion of the videos from the HVEC GoPro MP4 format to a compatible H264 MP4 format using the ffmpeg tool (open-source tool used for video processing) https://ffmpeg.org/ (accessed on 12 December 2022).
2. Segmentation and synchronization of the video footage from the six cameras. Segmentation involves cutting the videos using the ffmpeg tool. Camera synchronization is achieved by utilizing the extinction of external lighting as a synchronization event. The precise start and end frames of the events to be segmented are determined by observing three instances of lighting extinction before the event and one instance of lighting extinction after the event.
3. Camera calibration to correct for fish-eye effects. This step involves computing the internal and external parameters of the cameras to compensate for any distortion using the Matlab camera calibrator application https://fr.mathworks.com/help/vision/ug/using-the-single-camera-calibrator-app.html (accessed on 29 November 2022). An asymmetrical black and white checkerboard is used as a reference (as recommended in the Matlab user guide https://fr.mathworks.com/help/vision/ug/using-the-single-camera-calibrator-app.html, accessed on 29 November 2022), ensuring a variety of checkerboard views are captured by each camera.
4. Correction of the fish-eye effect in the videos using the intrinsic and extrinsic parameters obtained from the camera calibration step. The Matlab function undistortImage https://fr.mathworks.com/help/vision/ref/undistortimage.html (accessed on 29 November 2022) is employed for this purpose.

**Figure 5:**
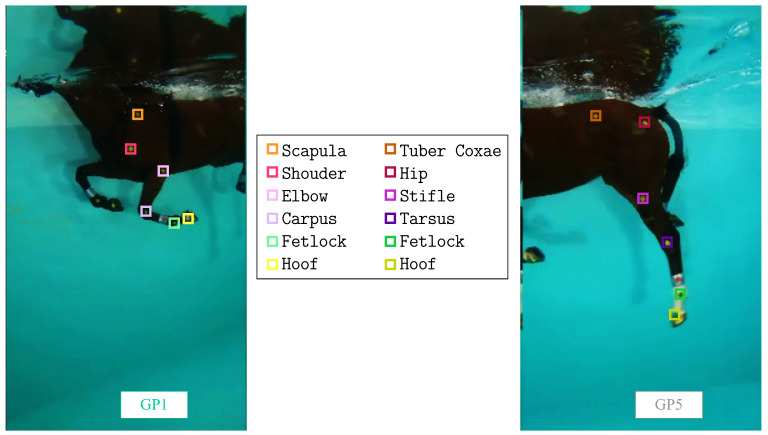
Example of tracking the 12 labels on the left side of the horse simultaneously from the videos captured by GoPro 1 and GoPro 5. **Left**: Front limb (markers from top to bottom: scapula, shoulder, elbow, carpus, front fetlock, and front hoof). **Right**: Hind limb (markers from top to bottom: tuber coxae, hip, stifle, tarsus, hind fetlock, and hind hoof).

5.1 Determination of the relative positioning of each pair of cameras. This step is performed using the Matlab Stereo camera calibrator and involves analyzing the checkerboard video footage to obtain the camera positioning for each camera pair. A minimum of 15 pairs of images with different checkerboard views are utilized for each camera pair.
5.2 Tracking of the anatomical points in the horse’s videos using the Matlab video labeler application https://fr.mathworks.com/help/vision/ug/get-started-with-the-video-labeler.html (accessed on 29 November 2022). This process involves automatic tracking (see Figure 5). In the event of automatic tracking failure, manual tracking is performed.

Upon completing the pre-processing steps, the obtained data consist of the 2D coordinates of the anatomical points of the horse captured in the videos, as well as the positioning information of the six cameras relative to each other. These data serve as the foundation for reconstructing the horse’s motion in different coordinate systems. By leveraging the 2D coordinates of the anatomical points, along with the known camera positions, it becomes possible to reconstruct the horse’s movement in three-dimensional space using the stereophotogrammetry method [4]. This reconstruction involves mapping the 2D coordinates onto a three-dimensional coordinate system, enabling the estimation of the horse’s position and motion in a more comprehensive manner.

### 2.3. Video Post-Processing

**Figure 6:**
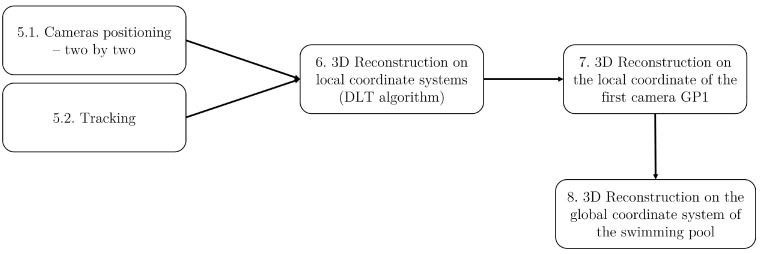
The three steps involved in post-processing the videos from the six GoPro cameras.

The video post-processing phase consists of three distinct steps (as illustrated in Figure 6), involving the reconstruction of the 3D coordinates in different coordinate systems:

6 The first step involves the 3D reconstruction of the anatomical points in the local coordinate system of each camera using the Direct Linear Transformation (DLT) algorithm [4]. By applying the DLT algorithm, along with the camera positioning data obtained during the pre-processing step (Step 5a), the 3D coordinates of the anatomical points of the horse are computed in the local coordinate system of the first camera within each camera pair (e.g., camera 3 for the 3–4 camera pair).
7 The subsequent step focuses on computing the 3D reconstruction in a unified coordinate system. Since there is an overlap in the field of view between each camera pair (as shown in Figure 1—bottom), the corresponding overlap is present in the 3D coordinates of the anatomical points. This enables the computation of a transition matrix that facilitates the transformation from one local coordinate system to another. Consequently, all the reconstructed points are transitioned from their respective local coordinate systems to the local coordinate system of the first GoPro camera.
8 The final step involves the computation of the 3D reconstruction in the global coordinate system of the swimming pool, as defined in Figure 2. In this coordinate system, the z-axis represents the vertical dimension (depicted in yellow), the y-axis represents the horizontal dimension (depicted in red), and the x-axis completes the trihedron. This global coordinate system provides a perspective of the 3D reconstruction as seen from a lateral viewpoint.

By following these three steps of video post-processing, the 3D reconstruction of the anatomical points of the horse is achieved in different coordinate systems, enabling a comprehensive analysis and visualization of the horse’s motion.

### 2.4. Error Estimation

**Figure 7:**
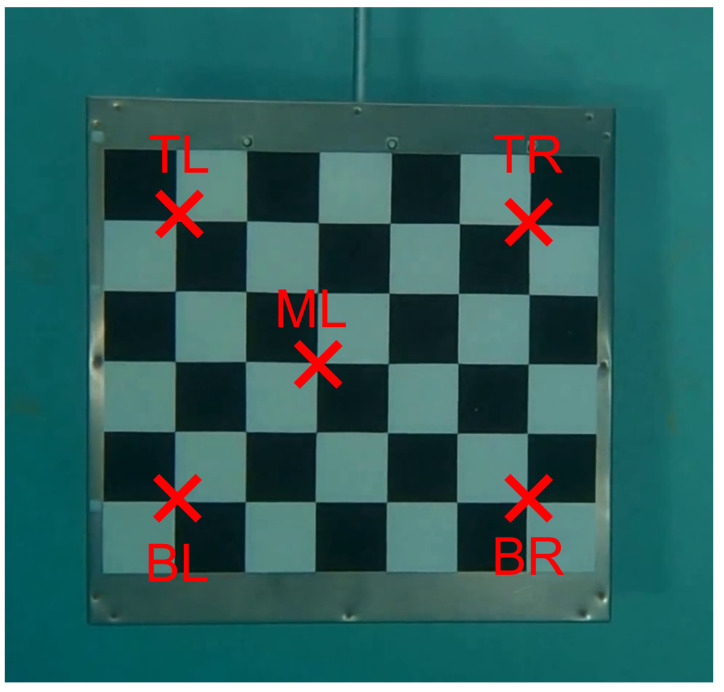
View of the checkerboard. Five specific points on the checkerboard are identified: TL = top left; TR = top right; ML = middle left; BL = bottom left; and BR = bottom right.

To estimate the error resulting from the entire pipeline, the 3D reconstruction of five specific points on the checkerboard (TL = top left, TR = top right, ML = middle left, BL = bottom left, and BR = bottom right) shown in Figure 7 is computed. These points have been selected to represent the range of lengths and angles that can be found in the anatomical segments and joint angles of the horse.

The 3D reconstruction is performed on a video of approximately a one-minute duration, during which the checkerboard passes in front of the six GoPro cameras. From this video, eight segments with lengths ranging from approximately 340 mm to 600 mm and five angles ranging from around 45° to 102° are computed using the 3D reconstructed points.

Table 2 provides details of the segments, including their lengths and corresponding labels. These segments are used to evaluate the accuracy of the 3D reconstruction by comparing the computed lengths to their actual values.

Similarly, Table 3 provides details of the angles, including their measurements and labels. These angles are used to assess the accuracy of the 3D reconstruction by comparing the computed angles to their real values.

### 2.5. Three-Dimensional Reconstruction of the Horse and Computation of Angles

**Figure 8:**
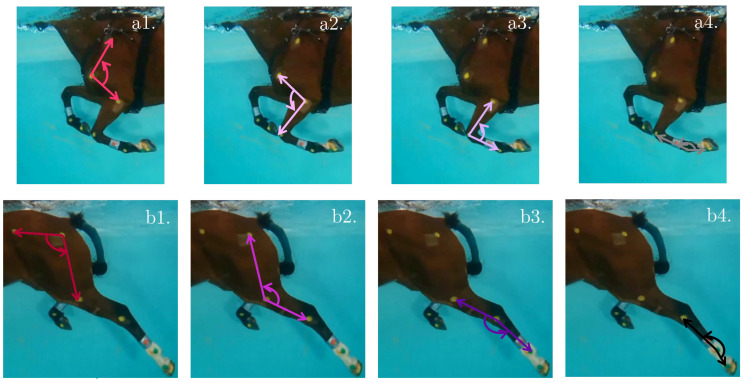
Reconstructed angles considered for the front limbs (light colors) and hind limbs (dark colors): (**a1**) Shoulder; (**a2**) Elbow; (**a3**) Carpus; (**a4**) Front fetlock; (**b1**) Hip; (**b2**) Stifle; (**b3**) Tarsus; and (**b4**) Hind fetlock.

The 3D reconstruction of the horse enables the computation of the anatomical joint angles, as depicted in Figure 8 and detailed in Table 4. These angles provide comprehensive information on different joint rotations in both the front and hind limbs of the horse.

For the front limb, the computed joint angles include:

1. Shoulder angle (Figure 8(a1)): This angle is formed between the scapula and the humerus, representing the flexion and extension of the shoulder joint.
2. Elbow angle (Figure 8(a2)): This angle is formed between the humerus and the radius, indicating the flexion and extension of the elbow joint.
3. Carpus angle (Figure 8(a3)): This angle is formed between the radius and the metacarpus, providing insights into the flexion and extension of the carpal joint.
4. Front fetlock angle (Figure 8(a4)): This angle is formed between the metacarpus and the digit of the forefoot, representing the flexion and extension of the fetlock joint in the front limb.

Similarly, for the hind limb, the following angles are considered:

1. Hip angle (Figure 8(b1)): This angle is formed between the ilium and the femur, indicating the flexion and extension of the hip joint.
2. Stifle angle (Figure 8(b2)): This angle is formed between the femur and the tibia, providing insights into the flexion and extension of the stifle joint.
3. Tarsus angle (Figure 8(b3)): This angle is formed between the tibia and the metatarsus, representing the flexion and extension of the tarsal joint.
4. Hind fetlock angle (Figure 8(b4)): This angle is formed between the metatarsus and the digit of the hindfoot, indicating the flexion and extension of the fetlock joint in the hind limb.

## 3. Results

### 3.1. Three-Dimensional Reconstruction—Error Estimation

**Figure 9:**
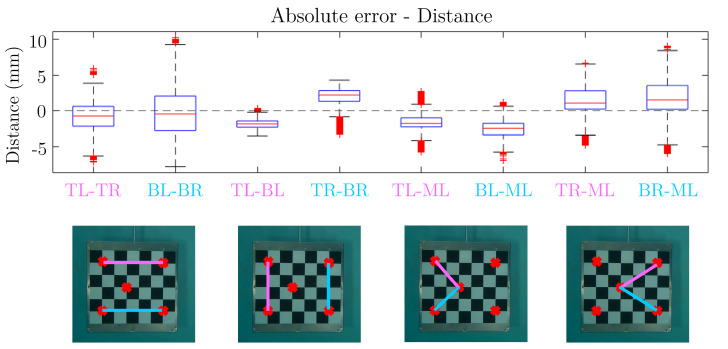
Error estimation of the segments of the checkerboard. From left to right: Horizontal lines TL-TR and BL-BR, vertical lines TL-BL and TR-BR, first diagonals TL-ML and BL-ML, and second diagonals TR-ML and BR-ML.

Figure 9 displays box plots depicting the absolute error between the computed values and the actual dimensions of the horizontal lines, vertical lines, first diagonals, and second diagonals during the entire one-minute video of the checkerboard. We can see that the absolute error is within a range of less than 10 mm, even for the largest segments. However, there are variations in precision among the different segments. We can see that the segments close to the horizontal lines are more dispersed and less precise compared to the segments close to the vertical lines, suggesting that there may be specific challenges or factors influencing the accuracy of horizontal segment reconstruction. Additionally, the less precise tracking of the bottom right point contributes to the variations in precision. Overall, despite these variations, the results demonstrate a precision on the order of centimeters.

**Figure 10:**
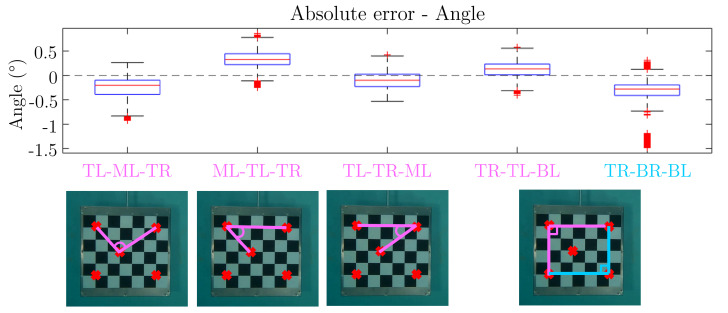
Error estimation of the angles of the checkerboard. From left to right: Corner triangle ML (TL-ML-TR), corner triangle TL (ML-TL-TR), corner triangle TR (TL-TR-ML), and corners of the checkerboard TL (TR-TL-BL) and BR (TR-BR-BL).

Figure 10 displays box plots representing the absolute error between the computed values and the actual angles of the corner triangles (ML, TL, TR) and the corners of the checkerboard (TL, BR) during the entire one-minute video of the checkerboard. The box plots demonstrate that for all reconstructed angles, the absolute error is within a range of less than 1°, even for the largest angles. It is worth noting that the corner checkerboard BR exhibits numerous outliers, which are attributed to the less precise reconstruction of the BR point. Despite this, the overall precision achieved for the reconstructed angles is on the order of a degree for angles measuring tens of degrees.

### 3.2. Three-Dimensional Reconstruction of Anatomical Segments of a Horse

**Figure 11:**
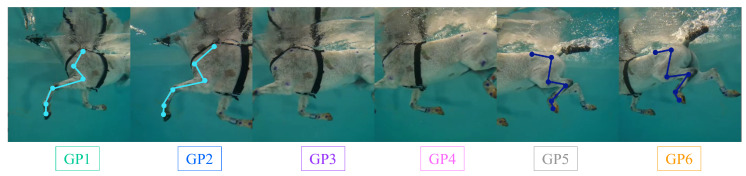
View from the 6 GoPro cameras and considered anatomical segments: light blue represents the front limb of the horse, and dark blue represents the hind limb of the horse. The same color code is used in the video of the 3D reconstruction, which can be found at https://zenodo.org/record/8365925 (accessed on 3 October 2023) (playable with VLC).

Figure 11 provides a visual representation of the six GoPro cameras capturing the horse at a specific moment when the entire horse is within the field of view. This viewpoint allows for the visualization of both the front and hind limbs from two different camera pairs: cameras 1–2 for the front limb and cameras 5–6 for the hind limb. These camera pairs are utilized to compute the 3D reconstruction of the anatomical points corresponding to the front and hind limbs simultaneously, as specified in Table 1. Based on the reconstructed anatomical points, the corresponding anatomical segments shown in Figure 11 are derived. For the front limb, the segments include the scapula, humerus, radius, metacarpus, and digit of the forefoot. On the other hand, for the hind limb, the segments comprise the ilium, femur, tibia, metatarsus, and digit of the hindfoot (Table 5). The video available at https://zenodo.org/record/8365925 (accessed on 3 October 2023) showcases the 3D reconstruction, offering a visual representation of the anatomical segments of the front (light blue) and hind (dark blue) limb reconstructions while the horse is in front of the GoPro cameras. It demonstrates that the 2.6 m field of view (Figure 1) effectively captures at least an entire swimming cycle for both the front and hind limbs of the horse.

### 3.3. Anatomical Joint Rotation

**Figure 12:**
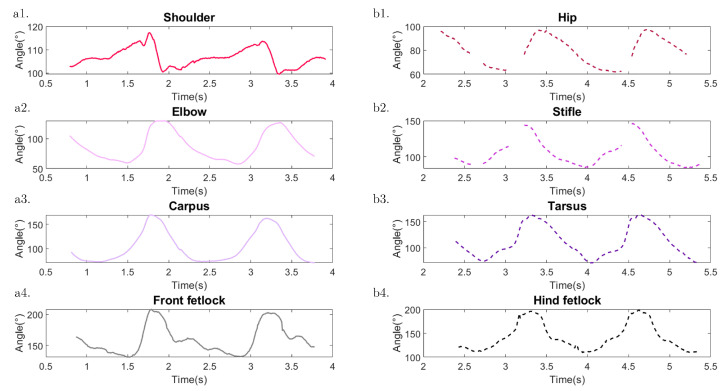
Example of reconstructed angles considered for the front limbs (light colors) and hind limbs (dark colors) over time. (**a1**) Shoulder; (**a2**) Elbow; (**a3**) Carpus; (**a4**) Front fetlock; (**b1**) Hip; (**b2**) Stifle; (**b3**) Tarsus; and (**b4**) Hind fetlock.

Figure 12 provides an example of the computed angles over time, specifically for the second swimming session of horse 8 (see Table A1 for the horse’s characteristics). The plot shows the variations in each reconstructed angle during the swimming motion. It is evident that two complete cycles of motion could be observed both for the front and hind limbs. Additionally, by analyzing the example, it is possible to estimate the duration of a swimming cycle for both the front and hind limbs, which is approximately 1.5 s.

Table 6 summarizes the values of the maximum flexion, maximum extension, and range of motion (ROM) for all horses (twelve videos were analyzed). For the front limb, the shoulder exhibits the smallest ROM, averaging 17°, with a maximum flexion of 102° and a maximum extension of 119°. On the other hand, the carpus shows the largest ROM, averaging 99°, with a maximum flexion of 69° and a maximum extension of 168°. Regarding the hind limb, the hip joint has the smallest ROM, averaging 39°, with a maximum flexion of 67° and a maximum extension of 107°. Conversely, the tarsus exhibits the largest ROM, averaging 99°, with a maximum flexion of 62° and a maximum extension of 162°.

## 4. Discussion

### 4.1. Underwater 3D MoCap

The proposed setup involved an acquisition system for underwater 3D motion capture and its application to horse swimming. First and foremost, it is a non-invasive system that does not disrupt or interfere with the natural swimming behavior of the horse. The modular aluminum structure enables a compact design, with a thickness of approximately 20 cm. This allows for unobtrusive placement in the swimming pool, ensuring that the horse remains comfortable and unaffected by the system’s presence. Another advantage is the affordability of the system. With a total cost of less than EUR 4000, it is a cost-effective solution that makes it accessible to veterinary clinics, research laboratories, and other institutions with limited budgets. This affordability enables the wider adoption and use of the system for studies and research involving 3D motion capture in aquatic environments. Furthermore, the system is robust and easy to install. Once the cameras are mounted on the aluminum structure, the entire system can be set up, including the foam phantom, field-of-view adjustment, and calibration, in approximately one hour. This ease of installation allows for efficient data collection during clinical studies, as the system can be quickly installed and uninstalled, leaving the swimming pool available for other purposes when not in use. Finally, the accuracy of the system, estimated using the checkerboard, leads to an accuracy of the order of centimeters for segments of tens of centimeters and of the order of a degree for angles. The non-invasiveness, affordability, and accuracy of the system make it suitable for use in the context of human swimming biomechanics evaluation [21]. The challenges of underwater filming are similar in both horse and human swimming, although in the case of humans, monitoring outside the water is also required.

In the context of 3D horse reconstruction, our presented pipeline relies on the use of animal marking sticks, which are composed of waxes, paraffin oil, and color pigments, to identify anatomical landmarks. This approach offers two distinct advantages over traditional spherical markers. Firstly, it avoids introducing additional drag. Secondly, it eliminates the need for adhesive glue, which can cause allergic reactions when attaching markers to the skin. However, it is essential that these markers are accurately placed in specific locations on the horse (Figure 3). This task requires either a veterinarian or an individual with a comprehensive understanding of horse anatomy to perform the drawing.

Finally, although the markers are appropriately positioned, not all of them are equally straightforward to track, potentially leading to the introduction of noise in the 3D reconstruction, and subsequently, in the computation of the joint angles. For instance, angles related to the fetlocks are more susceptible to noise compared to other angles. This is mainly due to the challenge of precisely tracking the foot, which undergoes out-of-plane rotations. Similarly, tracking the tuber coxae and hip joints can present difficulties when assessing the hip and stifle joints. The hip joint experiences substantial out-of-plane rotations, leading to situations where the tuber coxae and hip may be partially or entirely obscured by other parts of the limb. This obstruction hampers their visibility and accurate tracking.

**Figure A1:**
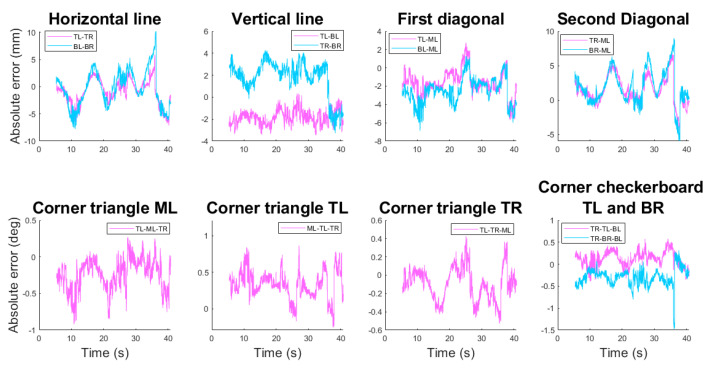
Computation of the measurement error of the checkerboard over time.

The pipeline we presented achieves a precision for the absolute error of less than 10mm and less than one degree for the checkerboard, aligning with findings in the literature related to underwater motion capture [28,29,30,31,32]. As mentioned in [30], it is worth noting that the results are improved when distortion is minimized, especially when employing underwater cameras. Evaluating the performance of the marker-based capture system involves analyzing the absolute error over time (Figure A1), which is an important aspect. Observations have shown that the most significant errors occur when the checkerboard undergoes high rotation around the z-axis, which corresponds to out-of-plane rotation within the cameras’ fields of view [33]. Consequently, this results in a greater out-of-plane error compared to the in-plane error. Considering the movement of the horse during swimming, which is mainly confined to the sagittal plane, it is expected that the precision obtained for anatomical segments would be around 10mm, and in terms of joint angles, the system provides precision in the order of a degree.

On land, the 3D reconstruction and computation of joint angles can aid in detecting lameness [34,35,36,37,38]. Comparing the joint angles of sound horses with those of horses with induced lameness can help in understanding the origin or automatically detecting lameness. For instance, the maximum fetlock angle appears to be a reliable indicator of forelimb lameness [37]. When examining the values, the mean value accuracy is around a tenth of a degree, but significant differences between sound horses and those with various lameness conditions are at least five degrees, within the same gait. Taking into account that swimming is a completely distinct gait and considering the precision of our system, one can envision utilizing specific angle values to determine the choice between earth training and swimming training for rehabilitation, for example. Overall, these findings demonstrate the suitability of the marker-based capture system and its associated post-processing methods for accurately capturing and quantifying the 3D movement of a horse’s anatomical points and joint angles during swimming activities.

### 4.2. Analysis of Horse Swimming

The system presented in this study allows the 3D reconstruction of the anatomical segments of horse limbs during a full cycle of swimming. These segments provide a detailed representation of the skeletal structure and allow for further analysis of the horse’s movement and kinematics during swimming.

To the best of our knowledge, this study is the first to obtain recordings from horses enabling the 3D reconstruction of an entire swimming cycle. This allows for the examination of lateral and diagonal limb coordination, as well as antero-posterior coordination. Describing the gait of a horse typically relies on certain events, such as the protraction and retraction of each limb. Both events can be detected for each limb in the videos, allowing for the future development of an indicator of the horse’s swimming quality as a follow-up measure in the rehabilitation or recovery process, akin to the use of the duty factor in land-based studies (e.g., [39]).

Furthermore, when examining the biomechanics of horses, the movement of their limbs is primarily influenced by the most proximal joints, namely the shoulder and the hip [40]. Additionally, in the case of horses, the main muscles that facilitate movement are located in the upper part (with only tendons and ligaments under the carpus and tarsus, which store elastic energy). To the best of our knowledge, this study is the first to record and analyze these two proximal joints during swimming. When comparing the results obtained for the maximal flexion and extension angles, as well as the range of motion (ROM), with the existing literature on horse swimming for the other three joints in each limb [24,25], the findings are consistent. However, several factors may account for the differences observed. Firstly, our study included a smaller sample size of six horses and twelve recordings, which was half the number of horses examined in the study conducted by Santosuosso et al. [24,25]. This could potentially have resulted in less diversity in the results. Secondly, in our study, the angles were measured in 3D, whereas Santosuosso et al. used 2D angles in their investigations [24,25]. This distinction may have introduced variations in the results, as the out-of-plane component was not accounted for in the 2D measurements, particularly for the hind limb, which undergoes out-of-plane rotation. Lastly, a significant difference lies in our capability to record movements underwater, whereas other studies [24,25] captured movements behind a glass window. The presence of a glass window introduces distortions in the light trajectory, resulting in less precise measurements. In our case, there was no distortion induced by the glass window; furthermore, image correction was achieved through the calibration process. Lastly, the precision of the reconstruction has been documented using the checkerboard. Overall, while there may be differences between our findings and those of previous studies, these variations can be attributed to factors such as sample size, measurement dimensionality, and the recording environment.

Upon closer examination of the joint angles, it is evident that all angles exhibit a greater range of motion during swimming compared to land-based training sessions (see Table A2 and Table A3 in Appendix C for values adapted from [24,25,27]) for both the front and hind limbs, except for the fetlock angles. The increased range of motion observed during swimming is consistently achieved through larger maximal flexion angles (smaller minimum angles) rather than larger maximal extension angles. In fact, for all gaits, the maximal extension angles are smaller for the front and hind limb angles, except for the hip and tarsus joints. The behavior of the fetlock angles is particularly relevant, as the maximal extension angles typically exceed 215° on land (up to 240° in canter) but do not exceed 205° in the swimming pool. Joint angles provide a valuable element to consider in guiding a veterinary team’s decision-making process for customizing an effective rehabilitation plan for an animal while also recognizing that the analysis of joint angle values and their range of motion during swimming remains an ongoing area of research. This research has the potential to contribute to the animal’s optimal recovery.

### 4.3. Limitations of the Process and Opportunities for Future Studies

The current semi-automatic pre-processing of the video described in Section 2.2 involves several manual tasks that can be time-consuming and limit the ease of collecting quantitative data within and between individuals. However, advancements in automation, particularly through the use of machine learning techniques, have the potential to alleviate these challenges and improve the efficiency of data collection and analysis [41,42]. Among the tasks that require significant time investment in the current workflow is the tracking of anatomical points. While tracking applications can offer some assistance, they may not always yield precise results due to factors such as water homogeneity, lighting conditions, or the complex nature of swimming motions. Training a neural network on annotated data to automate the tracking process has the potential to significantly enhance both efficiency and accuracy. By leveraging machine learning algorithms, the network can learn to track anatomical points reliably across different videos [42,43,44], reducing the need for manual intervention. By automating tasks like anatomical point tracking, the process of collecting quantitative data for the longitudinal monitoring of horses in swimming training could be greatly improved. However, it is important to note that the initial step toward training the neural network requires a substantial amount of labeled data, making semi-automatic tracking a necessary preliminary stage. In this context, our semi-automatically tracked data could serve as a training dataset for training a deep neural network. Additionally, the present study uses classic and well-established methods available in Matlab’s image processing toolbox. Future work could explore innovative techniques of photogrammetry [45] or tag recognition [46] to explore new opportunities to improve the proposed workflow through image segmentation or automatic marker recognition.

Finally, it is evident that errors can potentially arise when switching between camera pairs during the 3D reconstruction process, especially if certain anatomical points lack sufficient overlap between different camera pairs. While using multiple views from three or more cameras simultaneously would increase accuracy, it would also raise budgetary considerations and significantly extend the post-processing time. However, it is important to note that when calculating angles, as long as the three anatomical points involved in the computation are reconstructed from the same camera pair, this issue is mitigated. Therefore, a couple of cameras are sufficient for the 3D reconstruction of a horse.

## 5. Conclusions

In conclusion, our study presents an innovative methodology for low-cost 3D underwater motion capture and its application to horse swimming. The developed system offers a non-invasive, cost-effective, and easy-to-set-up solution for accurately tracking and reconstructing the 3D motion of large animals. By analyzing the swimming cycles of six horses, we obtained results consistent with the existing literature [24,25] and made additional contributions by simultaneously recording the movements of both the fore and hind limbs, as well as calculating the rotations of all anatomical joints, particularly the proximal joints.

These methods enable the computation of the joint range of motion, maximum flexion, and extension, providing valuable information for veterinarians in determining the suitability of swimming training for rehabilitation after an injury. Moreover, our system’s versatility makes it applicable not only to horses but also to other large animals and humans, offering a potential solution for motion capture in swimming scenarios, where existing alternatives may be expensive, complex to install, or detrimental to performance.

By introducing this original underwater motion capture pipeline, we contribute to the field of 3D reconstruction and motion analysis in large animals, opening up possibilities for further research and applications in various domains. This methodology holds promise for advancing the understanding of swimming biomechanics and facilitating effective rehabilitation strategies.

## Figures and Tables

**Table 1:** Anatomical markers on the front and hind limbs—from top to bottom.

N°	Front Limb (Pink Bubble)	Hind Limb (Yellow Bubble)
1	Scapular spine	Tuber coxae
2	Shoulder	Hip
3	Elbow	Stifle
4	Carpus	Tarsus
5	Front fetlock	Hind fetlock
6	Front hoof	Hind hoof

**Table 2:** Reconstructed segments of the checkerboard.

	Segment	Dimensions (in mm)
Horizontal Line	TL-TR and BL-BR	600.0
Vertical Line	TL-BL and TR-BR	480.0
First Diagonal	TL-ML and BL-ML	339.4
Second Diagonal	TR-ML and BR-ML	432.7

**Table 3:** Reconstructed angles of the checkerboard.

	Angle	Dimensions (in °)
Corner Triangle ML	TL-ML-TR	101.31
Corner Triangle TL	ML-TL-TR	33.69
Corner Triangle TR	TL-TR-ML	45.00
Corner Checkerboard TL and BR	TR-TL-BL and TR-BR-BL	90.00

**Table 4:** Reconstructed angles—from top to bottom.

N°	Front Limb (Light Colors)	Hind Limb (Dark Colors)
1	Shoulder	Hip
2	Elbow	Stifle
3	Carpus	Tarsus
4	Front fetlock	Hind fetlock

**Table 5:** Anatomical segments—from top to bottom.

N°	Front Limb (Light Blue)	Hind Limb (Dark Blue)
1	Scapula	Ilium
2	Humerus	Femur
3	Radius / Ulna	Tibia
4	Metacarpus	Metatarsus
5	Front digit	Hind digit

**Table 6:** Minimum and maximum values for the different angles and ranges of motion for the various joints (means and standard deviations). The means are based on the 12 recordings of the 6 horses (2 recordings per horse, 1 for the left limbs and 1 for the right limbs).

Joint	Maximal Flexion (in °)	Maximal Extension (in °)	ROM (in °)
Shoulder	102 ± 3	119 ± 5	17 ± 3
Elbow	58 ± 3	133 ± 9	76 ± 11
Carpus	69 ± 7	168 ± 7	99 ± 10
Front fetlock	129 ± 6	197 ± 11	68 ± 12
Hip	67 ± 7	107 ± 7	39 ± 3
Stifle	78 ± 5	146 ± 5	68 ± 7
Tarsus	62 ± 7	162 ± 7	99 ± 6
Hind fetlock	109 ± 6	203 ± 7	94 ± 6

## Data Availability

The raw data supporting the conclusions of this article will be made available by the authors, without undue reservation.

## Abbreviations

The following abbreviations are used in this manuscript:

GP	GoPro
DLT	Direct Linear Transformation
ROM	Range Of Motion
PSD	Proximal Suspensory Ligament Desmitis

## Appendix A. Horses’ Characteristics

Table A1 presents the main characteristics of the six horses.

**Table A1:** Horses’ characteristics.

Number	Gender	Age	Color
1	Mare	10	Chestnut
2	Mare	13	Bay
3	Gelding	11	Gray
4	Mare	10	Bay
6	Gelding	10	Chestnut
8	Gelding	10	Chestnut

## Appendix B. Accuracy of the Measurement

Figure A1 presents the absolute error over time in millimeters for the eight distances and in degrees for the five angles considered. The peaks correspond to the large out-of-plane angles of the checkerboard.

## Appendix C. Comparison of the Angles of the Horses’ Limbs During Swimming and on Land

Table A2 and Table A3 present a comparison of the angles in the swimming pool and on land for walking, trotting, and cantering for the fore and hind limbs. The values during swimming are from this paper and the work by Santosuosso et al. [24,25], whereas the values when on land are from the work by Back et al. [27].

**Table A2:** Comparison of the angles of the fore limbs during swimming and on land.

Fore Limb	Our Study	Santosuosso et al. [24]	Walking [27]	Trotting [27]	Cantering (Trailing Fore Limb) [27]	Cantering (Leading Fore Limb) [27]
**Shoulder**						
Maximal flexion (in°)	102 ± 3		113.1 ± 4.2	110.1 ± 5.1		
Maximal extension (in°)	119 ± 5		126.2 ± 5.9	125.4 ± 4.2		
ROM (in°)	17 ± 3		13.1 ± 1.9	15.3 ± 1.8		
**Elbow**						
Maximal flexion (in°)	58 ± 3	52.8 ± 12.9	104.8 ± 3.6	95.3 ± 4.3	98.1 ± 4.6	88.8 ± 6.7
Maximal extension (in°)	133 ± 9	125.9 ± 15.7	156.6 ± 3.1	155.5 ± 3.1	159.9 ± 2.1	156.5 ± 3.8
ROM (in°)	76 ± 11	73.1 ±16.5	51.8 ± 3.6	60.2 ± 4.0	61.8 ± 4.8	67.7 ±5.6
**Carpus**						
Maximal flexion (in°)	69 ± 7	60.9 ± 7.0	110.1 ± 6.2	92.1 ± 7.4	97.0 ± 7.9	88.8 ± 8.3
Maximal extension (in°)	168 ± 7	156.7 ± 12.3	178.7 ± 2.3	182.9 ± 2.6	186.7 ± 3.6	184.4 ± 2.8
ROM (in°)	99 ± 10	98.5 ± 16.6	68.7 ± 5.7	90.8 ± 7.1	89.7 ± 7.0	95.6 ± 8.1
**Fetlock**						
Maximal flexion (in°)	129 ± 6	111.4 ± 9.1	163.5 ±6.4	158.7 ± 6.8	157.2 ± 7.3	150.9 ± 6.1
Maximal extension (in°)	197 ± 11	188.3 ± 5.3	221.1 ± 6.1	253.9 ± 6.2	242.4 ± 4.7	239.0 ± 8.1
ROM (in°)	68 ± 12	69.9 ± 9.7	60.2 ± 1.4	80.6 ± 7.1	85.2 ± 8.0	88.1 ± 7.5

**Table A3:** Comparison of the angles of the hind limbs during swimming and on land.

Hind Limb	Our Study	Santosuosso et al. [25]	Walking [27]	Trotting [27]	Cantering (Trailing Hind Limb) [27]	Cantering (Leading Hind Limb) [27]
**Hip**						
Maximal flexion (in°)	67 ± 7		86.3 ±3.2	88.9 ± 3.6	89.0 ± 3.2	83.7 ± 3.6
Maximal extension (in°)	107 ± 7		111.1 ± 3.8	112.2 ± 3.9	113.3 ± 3.2	106.2 ± 4.4
ROM (in°)	39 ± 3		24.8 ± 1.9	23.3 ± 1.8	24.3 ± 1.8	22.5 ± 1.9
**Stifle**						
Maximal flexion (in°)	78 ± 5	60.5 ± 8.3	133.9 ± 5.9	121.7 ± 5.1	124.5 ± 4.3	122.1 ± 3.0
Maximal extension (in°)	146 ± 5	111.5 ± 17.1	173.0 ± 4.9	169.0 ± 4.0	166.5 ± 5.1	177.7 ± 4.6
ROM (in°)	68 ± 7	50.9 ± 17.3	39.1 ± 3.7	47.3 ± 3.8	42.0 ± 4.7	55.5 ± 3.9
**Tarsus**						
Maximal flexion (in°)	62 ± 7	43.7 ± 5.1	134.0 ± 0.1	114.3 ± 5.1	124 ± 8.6	115.5 ± 8.0
Maximal extension (in°)	162 ± 7	154.7 ± 4.1	169.6 ± 3.5	169.7 ± 2.9	173.0 ± 3.0	171.9 ± 2.6
ROM (in°)	99 ± 6	110.7 ± 4.8	35.6 ± 4.5	55.4 ± 5.3	49.0 ± 7.6	56.5 ± 8.3
**Fetlock**						
Maximal flexion (in°)	109 ± 6	76.8 ± 5.7	145.2 ± 9.1	148.0 ± 7.5	144.4 ± 7.3	136.2 ± 8.9
Maximal extension (in°)	203 ± 7	188.9 ± 5.98	217.0 ± 4.2	233.0 ± 4.0	240.6 ± 3.8	233.9 ± 3.4
ROM (in°)	94 ± 6	112.2 ± 10.1	71.8 ± 9.0	85.0 ± 7.7	96.1 ± 7.0	97.7 ± 9.1
